# The Supplementation of FloraMax-B11 Did Not Affect the Bile Acid Neosynthesis and the Enterohepatic Circulation in Broiler Chickens

**DOI:** 10.3390/ani12212901

**Published:** 2022-10-22

**Authors:** Kouassi R. Kpodo, Atul Chaudhari, Lori L. Schreier, Katarzyna B. Miska, Monika Proszkowiec-Weglarz

**Affiliations:** 1 Animal Biosciences and Biotechnology Laboratory, United States Department of Agriculture, Agricultural Research Service, Beltsville, MD 20705, USA; 2 Oak Ridge Institute for Science and Education, Oak Ridge, TN 37830, USA

**Keywords:** probiotic, bile acid, enterohepatic circulation, broiler chickens

## Abstract

**Simple Summary:**

Most probiotic bacteria used in the poultry industry have bile salt hydrolase enzymes that can deconjugate bile salts resulting in bile acid excretion and potential impairment of bile salt functions in fat digestion and absorption. Whether probiotics affect bile acid metabolism in chickens is not well understood. In this study, chickens were given a probiotic following a specific dosage timing (3-, 10-, 21-, and 35-days post-hatch) or an antibiotic growth promoter for 35 days post-hatch. Various genes involved in bile acid synthesis and transport, as well as ileal deoxycholic acid and plasma cholic acid levels, were determined. Most of the genes, ileal deoxycholic acid, and plasma cholic acid, were not affected by the probiotic supplementation, but all the genes were affected by age. These results suggest that the probiotics did not affect bile acid neosynthesis and enterohepatic circulation but were age dependent. The data in this study may help improve probiotic usage as an antibiotic alternative in broiler chickens.

**Abstract:**

Most probiotics possess bile salt hydrolase enzymes and may increase bile acid excretion and negatively affect fat digestion and absorption. Therefore, the study objective was to determine the time course effects of a commercial probiotic (P) FloraMax-B11 (FM) supplementation on bile acid neosynthesis and enterohepatic circulation in broiler chickens. Fertile Ross 708 eggs were incubated under standard commercial conditions. At hatch, chicks (*n* = 550) were randomly assigned to 5 treatment groups (*n* = 5 replicates per treatment group) with 22 birds per pen. The 5 treatment groups consisted of: control group (C, normal water from hatch to 35 days of age without supplements); P3, water supplemented with FM for the first 3 days post-hatch followed by normal water until day 35; P10, water supplemented with FM for the first 10 days post-hatch followed by normal water until day 35; P35, water supplemented with FM from hatch to day 35; and AGP, water supplemented with antibiotic growth promoter (AGP) from hatch until day 35. Ileum, liver, and plasma were collected at hatch, days 3, 10, 21, and 35 post-hatch. The relative mRNA expression of genes involved in bile acid synthesis (CYP7A1, CYP8B1, FXR, FGFR4, and FGF19) and transport (ASBT, I-BABP, OSTα, OSTβ, and BSEP) as well as ileal deoxycholic acid and plasma cholic acid were determined. There was no FM and AGP interaction for any of the response criteria. No FM or AGP effects were observed (*p* > 0.05) for any genes, except FGF19, which expression was increased (*p* < 0.0001) in AGP compared to P35. No FM or AGP effects were observed (*p* > 0.05) for levels of deoxycholic and cholic acids. However, all the genes, deoxycholic acid, and plasma cholic acid were affected by age (*p* < 0.0001). In general, the data indicate that FM did not negatively impact bile acid metabolism and enterohepatic circulation, which appeared to be age dependent. However, more research should be conducted to confirm these results and investigate the effects of FM on bile acid metabolism, fat digestion, and intestinal microbiota in broiler chickens.

## 1. Introduction

Intestinal health is associated with its microbiota, which plays an important role in maintaining physiological homeostasis in the gut [1]. Because the intestinal microbiota is crucial in providing defense mechanisms against pathogens, they have gained attention as potential alternatives to antibiotic growth promoters (AGPs). Probiotics are bacteria that, when given at the right amount, provide health benefits to the host [2]. They have been shown to reduce necrotic enteritis and improve production performance in poultry [3,4]. The protective functions of probiotics include competition with pathogenic bacteria for mucosal surface and nutrients, enhancement of epithelial barrier functions by stimulating intestinal mucus layer production and promoting the development of the host innate immune system, and secretion of antimicrobial peptides and toxins [5]. In addition to the protective functions, probiotics are involved in metabolism through the fermentation of undigested dietary fibers and proteins, and the deconjugation of bile salts [6]. 

Bile acids are produced in the liver, conjugated mostly with taurine into bile salts in chickens, and stored in the gallbladder during the postprandial state [7]. When feed is consumed, the hormone cholecystokinin causes the contraction of the gallbladder resulting in the release of bile salts into the intestine where they facilitate fat digestion and absorption of end products such as fatty acids and monoglycerides as well as the absorption of fat-soluble vitamins [7,8]. About 95% of the bile acids are reabsorbed along the intestine and recycled to maintain the bile acid pool through the enterohepatic circulation [7]. The remaining 5% is lost in the excreta or feces and is compensated by de novo bile acids synthesis in the liver [7]. The excretion of bile acids is facilitated by the activity of the bacteria bile salt hydrolase enzyme. This enzyme deconjugates bile salts to their secondary forms, which are readily excreted. The deconjugation of bile salts in the intestine reduces the bile acid pool and may impair fat digestion and absorption, and production performance [9].

Bile salt hydrolase activity can be reduced by AGP usage. Previous studies have shown that AGPs significantly reduced *Lactobacillus* species [10,11,12], and this may subsequently reduce bile salt hydrolase activities. The reduction of bile salt hydrolase has been linked to the AGP-induced improvement of production performance [13]. Because of the ban of AGPs from chicken feed, the poultry industry is relying more on probiotics as alternatives to AGPs. Paradoxically, lactic acid-producing bacteria generally used as probiotics in chickens have bile salt hydrolase enzyme and can deconjugate bile salts [10,14]. Therefore, it is necessary to further understand how these probiotics impact bile acid metabolism.

FloraMax-B11 (FM) is a commercial lactic acid-based probiotic made of *Lactobacillus salivarius* and *Pediococcus parvulus.* It has been shown to improve production parameters [15,16], reduce the colonization of chickens with pathogenic bacteria, and mitigate the negative impacts of enteric diseases [17,18]. Provided that lactic acid-producing bacteria have bile salt hydrolase activities [19]; how FM affects the bile acid metabolism and enterohepatic circulation in chickens is not well understood. Therefore, the objective of this experiment was to determine the effects of different dosing regimens of FM supplementation on bile acid metabolism and enterohepatic circulation in broiler chickens. We hypothesized that FM would increase bile salt deconjugation and bile acid excretion, thereby increasing bile acid neosynthesis in the liver post-hatch.

## 2. Materials and Methods

### 2.1. Animals and Experimental Protocol

All animal procedures used in this experiment were approved by the Animal Care and Use Committee (protocol no. 19-003) of the Beltsville Animal Agricultural Research Center (BARC), in Beltsville, Maryland. Fertile Ross 708 eggs (*n* = 650) were purchased from Longenecker’s hatchery (Elizabethtown, PA, USA) and set in a Natureform incubator (Hawkhead Hatchery Equipment, Orange Park, FL, USA) under standard commercial conditions (99.5 °C and 60% RH). Eggs were candled at day 10 of the embryonic development, and non-fertile eggs were discarded. At hatch, chicks (*n* = 550) were randomly assigned to 5 treatment groups (*n* = 5 replicates per treatment group) with 22 birds per replicate. The 5 treatment groups consisted of control (C, normal water from hatch to day 35 of age without supplements); probiotic (P)3, water supplemented with FM for the first 3 days post-hatch followed by normal water until day 35; P10, water supplemented with FM for the first 10 days post-hatch followed by normal water until day 35; P35, water supplemented with FM from hatch to day 35; and AGP, water supplemented with AGP from hatch until day 35. Because AGPs are phased out of chicken feed due to antibiotic resistance and transmission to consumer’s diets, it is important to further understand the use of probiotics as antibiotic alternatives. Therefore, the AGP treatment was included in the experiment as a positive control.

The AGP used was Bacitracin Methylene Disalicylate (BMD 50 Soluble, Alpharma, Inc., Fort Lee, NJ, USA) at a recommended dose of 50 g/ton of feed by the manufacturer. FloraMax B-11^®^ (Pacific Vet Group, Fayetteville, AR, USA) was delivered in the drinking water following the manufacturer’s recommendations (1 bottle of 140 g of FM per 20,000 birds: 10^6^ cfu/mL of drinking water [15,18]), and water was supplied in bucket waterers equipped with 4 nipple drinkers each. Birds were housed in floor pens covered with wood shavings from hatch to 35 days of age. All birds had at *libitum* access to feed and water and were fed a commercial corn-soybean-based diet formulated to meet or exceed the NRC requirements. From hatch to day 21, birds were fed a starter diet containing 23% crude protein, 3000 kcal/kg metabolizable energy, 5.6% fat, 1% calcium, and 0.77% phosphorus. From day 22 to 35, birds were fed a grower diet containing 21% crude protein, 3100 kcal/kg metabolizable energy, 5.8% fat, 0.81% calcium, and 0.66% phosphorus. Birds were euthanized for tissue collection at hatch (*n* = 6) and on days 3, 10, 21, and 35 post-hatch (*n* = 5 per treatment group for each day: 20 in total). Ileal tissue (1 cm in the middle of the distal part) was excised, the content gently pressed into collection tubes, and snap-frozen in liquid nitrogen. The same ileal section was opened longitudinally and spread on a cutting board, and the mucosa was scraped using a glass microscope slide. The scrapings were collected in tubes and snap-frozen in liquid nitrogen. Liver samples were collected and snap-frozen in liquid nitrogen. Blood samples were collected in heparinized tubes by heart puncture immediately following euthanasia. Plasma was harvested after centrifugation at 1900× *g* and 4 °C for 15 min, aliquoted and stored at −20 °C.

### 2.2. RNA Extraction and Reverse Transcription-Quantitative PCR

Total RNA extraction, complementary DNA (cDNA) synthesis, and real-time qPCR were performed as previously described [20]. Briefly, total RNA was extracted from mucosal scrapings and liver samples using the RNeasy Mini QIAcube kit and QIAcube instrument (Qiagen, Valencia, CA, USA) following the manufacturer’s protocols. The quality of the resulting RNA was evaluated on a NanoDrop One (ThermoFisher Scientific, Inc., Waltham, MA, USA), and 0.5 µg of the RNA was reverse transcribed to cDNA using the Superscript IV reverse transcriptase (Invitrogen, Carlsbad, CA, USA) and Oligo dT primers following the manufacturer’s instructions. The cDNA was diluted 1:10 and used in the real-time PCR reactions, which were performed in 15 µL total volume containing 2 µL of cDNA, 400 nM of reverse and forward of each gene-specific primer, and SsoAdvanced Universal SYBR Green Supermix (Bio-Rad, Hercules, CA, USA) using the CFX96TM Touch System (Bio-Rad). The thermal cycling parameters were set at 95 °C for 5 min followed by 40 cycles of 95 °C for 15 s, 60 °C for 30 s, and 72 °C for 30 s. A Melt curve analysis was performed at the end at 95 °C, and gel electrophoresis was run on the qPCR products to ensure that the amplicon was the appropriate size and only the genes of interest were amplified. Except for the housekeeping genes, primer sequences of all the genes were designed using the Primer3 software [21], and primer sequences and accession numbers are presented in Table 1. Primers for housekeeping genes glyceraldehyde-3-phosphate dehydrogenase (GAPDH), beta-actin (β-actin), and beta2-microglobulin (β2-m) were previously published [20]. Ileal samples were analyzed for apical sodium-dependent bile acid transporter (ASBT), ileal bile acid binding protein (I-BABP), farnesoid X receptor (FXR), organic solute transporter alpha (OSTα), organic solute transporter beta (OSTβ), and fibroblast growth factor 19 (FGF19). Liver samples were analyzed for cholesterol 7 alpha-hydroxylase (CYP7A1), sterol 12 alpha-hydroxylase (CYP8B1), small heterodimer partner (SHP), fibroblast growth factor receptor 4 (FGFR4), beta klotho (βklotho), bile salt exporter pump (BSEP), and FXR. The data were normalized to the geometric mean of the 3 housekeeping genes (GAPDH, β-actin, and β2-m), and relative gene expression was calculated using the 2^-ΔΔCt^ method [22]. The data were analyzed and presented as fold change relative to the hatch (d 0) group.

### 2.3. Ileal Deoxycholic and Plasma Cholic Acid Analysis

For deoxycholic acid, ileal content (50 mg) was homogenized in 2 mL of ice-cold PBS using an Omni TH^TM^ homogenizer (Omni International, Kennesaw, GA, USA). The homogenate was incubated at 4 °C for 20 min, then transferred to a microcentrifuge tube, and centrifuged at 12,000× *g* for 20 min. The supernatant was transferred to a fresh microcentrifuge tube and stored at −20 °C for deoxycholic acid analysis. Deoxycholic acid was analyzed using a commercially available ELISA kit (Deoxycholic acid; LSBio, Seattle, WA, USA) following the manufacturer’s instructions. The homogenates were either diluted 1:10, 1:20, or 1:40.

For cholic acid, plasma samples were diluted 1:10, and the cholic acid level was determined using a commercially available ELISA kit (cholic acid; Cell Biolabs, San Diego, CA, USA) following the manufacturer’s instructions.

The concentrations of both deoxycholic acid and cholic acid were measured at 450 nm absorbance on the plate reader (SpectraMax M2, Molecular Devices, San Jose, CA, USA), and the results were calculated from a standard curve determined for each plate using the SpectraMax M2 software.

### 2.4. Statistical Analysis

Gene expression, cholic acid, and deoxycholic acid data were analyzed using the GLIMMIX procedure (SAS 9.4, Cary, NC, USA). Data were log-transformed to meet assumptions of normality when needed, and untransformed least squares means were reported. Age (d post-hatch) and treatments (C, P3, P10, P35, and AGP) and their interaction were set as fixed effects. However, the interaction was not significant for any analyses and was removed from the model; therefore, only the main effects are reported. The plate was set as a random factor for cholic acid and deoxycholic acid but was not significant for any analyses and was removed from the model. Means were separated using Tukey’s adjustment. Statistical significance was set at *p* < 0.05, and tendency was considered at 0.05 < *p* < 0.10.

## 3. Results

### 3.1. Gene Expression

#### 3.1.1. Ileal Gene Expression

**Figure 1:**
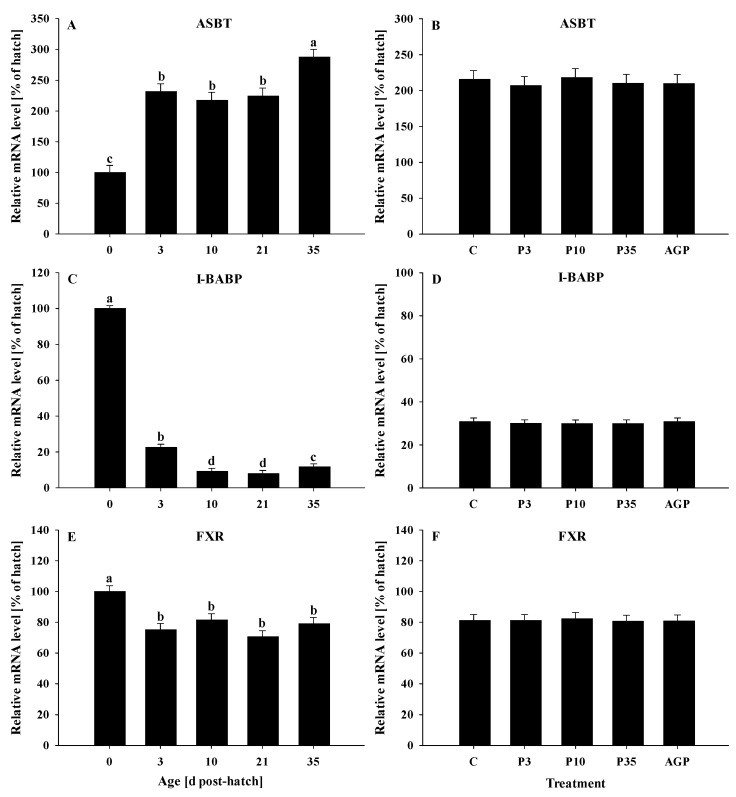
Age and treatment effects on mRNA levels of genes associated with bile acid metabolism in the ileum. Age effects on (**A**) apical sodium-dependent bile acid transporter (ASBT), (**C**) ileal bile acid binding protein (I-BABP), and (**E**) farnesoid X receptor (FXR). Treatment effects on (**B**) ASBT, (**D**) I-BABP, and (**F**) FXR. The expression level of hatch (day 0) was set to 100%, and other values were calculated as a % of the hatch data. Each value represents mean ± SE. Treatments are C, normal water (without supplements) from day 0 to 35 post-hatch; P3, FM in water for first 3 days post-hatch, followed by normal water until day 35; P10, FM in water for first 10 days post-hatch, followed by normal water until day 35; P35, FM in water from hatch to day 35; and AGP, AGP in water from hatch to day 35. **^a,b,c,d^** Letters indicate significance (*p* < 0.05) between days post-hatch.

All genes were significantly (*p* < 0.05) affected by age. The ASBT mRNA expression was increased (*p* < 0.0001) from day 0 to 3, remained constant at days 3, 10, and 21, but was significantly increased at day 35 (Figure 1A). No treatment differences were observed (*p* = 0.97) for ASBT mRNA expression (Figure 1B).

I-BABP mRNA expression was significantly reduced (*p* < 0.0001) from day 0 to 3 and again at day 10, remained constant at days 10 and 21, followed by a slight increase at day 35; however, the I-BABP mRNA level at day 35 was lower compared to that of day 3 (Figure 1C). No treatment differences were observed (*p* > 0.57) for I-BABP expression (Figure 1D).

The FXR mRNA expression was reduced (*p* < 0.0001) from day 0 to 3 and remained constant until day 35 post-hatch (Figure 1E). No treatment differences were observed (*p* = 0.73) for FXR mRNA expression (Figure 1F).

**Figure 2:**
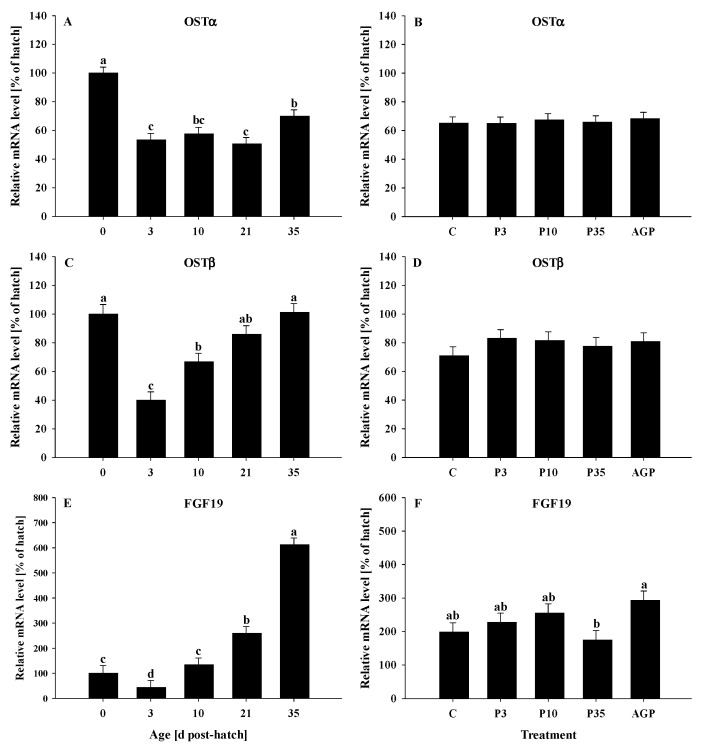
Age and treatment effects on mRNA levels of genes associated with bile acid metabolism in the ileum. Age effects on (**A**) organic solute transporter alpha (OSTα), (**C**) organic solute transporter beta (OSTβ), and (**E**) fibroblast growth factor 19 (FGF19). Treatment effects on (**B**) OSTα, (**D**) OSTβ, and (**F**) FGF19. The expression level of hatch (day 0) was set to 100%, and other values were calculated as a % of the hatch data. Each value represents mean ± SE. Treatments are C, normal water (without supplements) from days 0 to 35 post-hatch; P3, FM in water for first 3 days post-hatch, followed by normal water until day 35; P10, FM in water for first 10 days post-hatch, followed by normal water until day 35; P35, FM in water from hatch to day 35; and AGP, AGP in water from hatch to day 35. **^a,b,c,d^** Letters indicate significance (*p* < 0.05) between days post-hatch or treatments.

The mRNA expression of OSTα was reduced (*p* < 0.0001) from day 0 to 3, remained constant from day 3 to 21, then increased at day 35; but no OSTα mRNA expression differences were observed between days 10 and 35 (Figure 2A). No treatment differences were observed (*p* > 0.94) for OSTα mRNA expression (Figure 2B).

The mRNA expression of OSTβ was reduced (*p* < 0.0001) from day 0 to 3, then gradually increased at day 10, and returned to day 0 levels at days 21 and 35 (Figure 2C). No treatment differences were observed (*p* = 0.59) for OSTβ mRNA expression (Figure 2D).

The mRNA expression of FGF19 was reduced (*p* < 0.0001) from day 0 to 3 and then was increased gradually from day 10 to its highest level at day 35; however, no differences were observed in mRNA expression between days 0 and 10 (Figure 2E). The mRNA expression of FGF19 was increased (*p* < 0.05) in AGP compared to P35 birds (Figure 2F). No other treatment differences were observed for the FGF19 mRNA expression (Figure 2F).

#### 3.1.2. Liver Gene Expression

**Figure 3:**
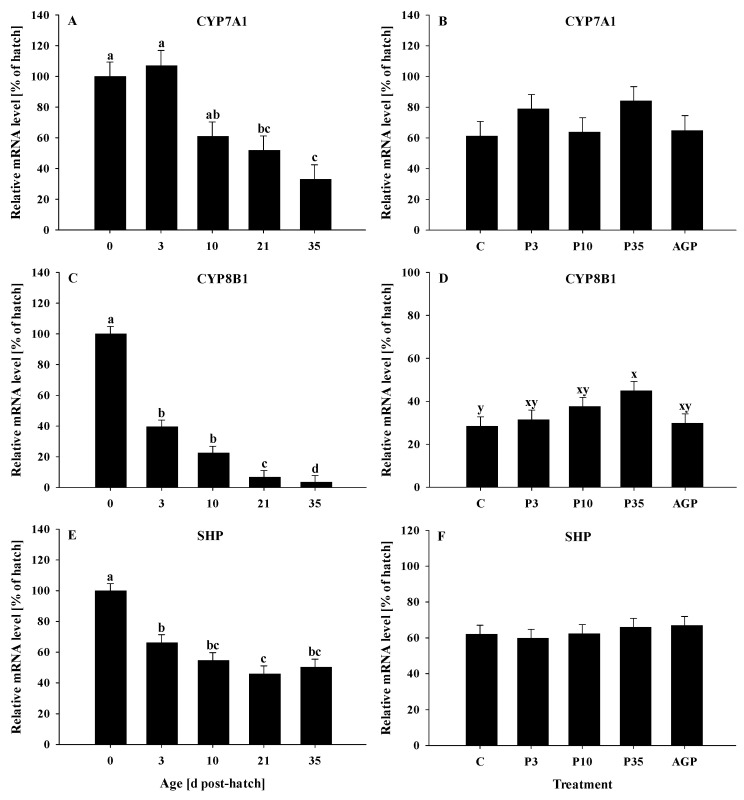
Age and treatment effects on mRNA levels of genes associated with bile acid metabolism in the liver. Age effects on (**A**) cholesterol 7α-hydroxylase (CYP7A1), (**C**) sterol 12 alpha-hydroxylase (CYP8B1), and (**E**) small heterodimer partner (SHP). Treatment effects on (**B**) CYP7A1, (**D**) CYP8B1, and (**F**) SHP. The expression level of hatch (day 0) was set to 100%, and other values were calculated as a % of the hatch data. Each value represents mean ± SE. Treatments are C, normal water (without supplements) from days 0 to 35 post-hatch; P3, FM in water for first 3 days post-hatch followed by normal water until day 35; P10, FM in water for first 10 days post-hatch followed by normal water until day 35; P35, FM in water from hatch to day 35; and AGP, AGP in water from hatch to day 35. **^a,b,c,d^** Letters indicate significance (*p* < 0.05) between days post-hatch. **^x,y^** Letters indicate tendency (0.05 < *p* < 0.10) between treatments.

All genes were significantly (*p* < 0.0001) affected by age. The mRNA expression of CYP7A1 was constant at days 0, 3, and 10 and decreased at days 21 and 35, but no differences were observed between days 10 and 21 or between days 21 and 35 (Figure 3A). No treatment differences were observed (*p* = 0.66) for the mRNA expression of CYP7A1 (Figure 3B).

The mRNA expression of CYP8B1 was reduced (*p* < 0.0001) from day 0 to 3, remained constant between days 3 and 10, then decreased at day 21 and reached its lowest value at day 35 (Figure 3C). The mRNA expression of CYP8B1 tended (*p* = 0.07) to be increased in P35 compared to C (Figure 3D). No other treatment differences were observed for the mRNA expression of CYP8B1 (Figure 3D).

The mRNA expression of SHP was reduced (*p* < 0.0001) from day 0 to 3 and remained lower the rest of the days; however, it was reduced at day 21 compared to days 0 and 3 (Figure 3E). No treatment effects were observed (*p* = 0.78) for SHP mRNA expression (Figure 3F).

**Figure 4:**
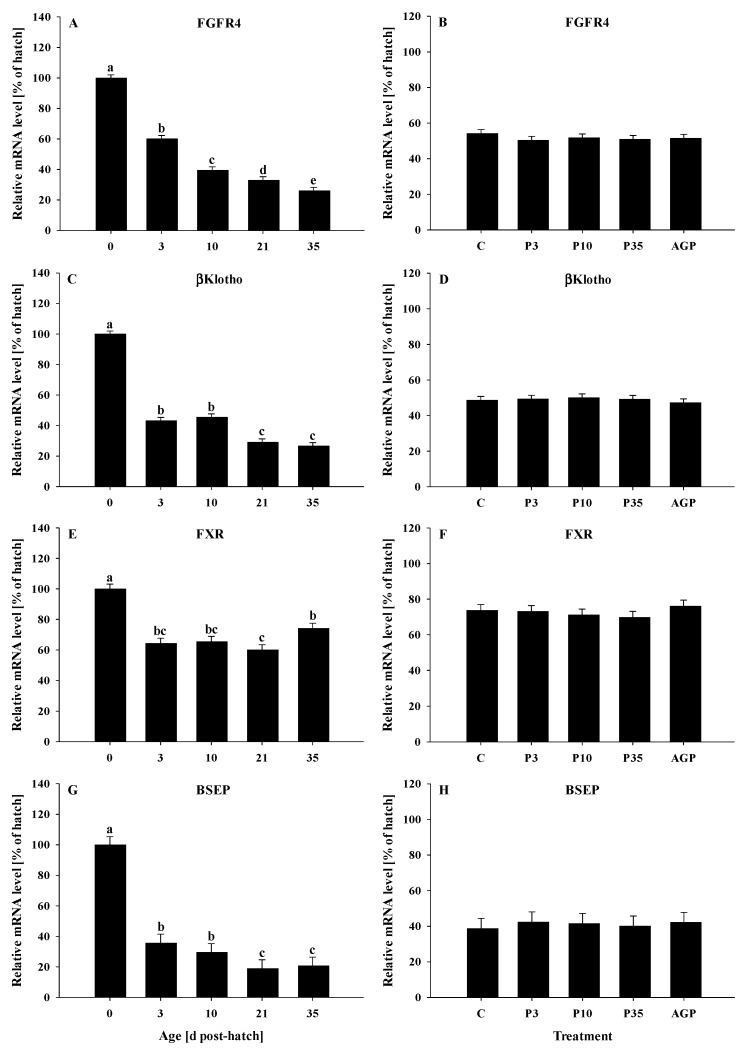
Age and treatment effects on mRNA levels of genes associated with bile acid metabolism in the liver. Age effects on (**A**) fibroblast growth factor receptor 4 (FGFR4), (**C**) beta klotho (βklotho), (**E**) farnesoid X receptor (FXR), and (**G**) bile salt exporter pump (BSEP). Treatment effects on (**B**) FGFR4, (**D**) βklotho, (**F**) FXR, and (**H**) BSEP. The expression level of hatch (day 0) was set to 100%, and other values were calculated as a % of the hatch data. Each value represents mean ± SE. Treatments are C, normal water (without supplements) from day 0 to 35 post-hatch; P3, FM in water for first 3 days post-hatch, followed by normal water until day 35; P10, FM in water for first 10 days post-hatch, followed by normal water until day 35; P35, FM in water from hatch to day 35; and AGP, AGP in water from hatch to day 35. **^a,b,c,d,e^** Letters indicate significance (*p* < 0.05) between days post-hatch.

The mRNA expression of FGFR4 decreased (*p* < 0.0001) gradually from day 0 to 35 (Figure 4A). No treatment differences were observed (*p* = 0.69) for the mRNA expression of FGFR4 (Figure 4B).

The mRNA expression of βklotho was reduced (*p* < 0.0001) from day 0 to 3, remained constant at days 3 and 10, then was reduced to the same levels at days 21 and 35 (Figure 4C). No treatment effects were observed (*p* = 0.96) for the mRNA expression of βklotho (Figure 4D).

The mRNA expression of FXR was reduced (*p* < 0.0001) from day 0 to 3 and remained constant at days 10 and 21, and then increased slightly at day 35; however, the mRNA expression of FXR was reduced at day 21 compared to day 35. In addition, the mRNA expression of FXR was lower at day 35 compared to day 0 (Figure 4E). No treatment differences were observed (*p* = 0.73) for mRNA expression of FXR (Figure 4F).

The mRNA expression of BSEP was reduced (*p* < 0.0001) from day 0 to 3, remained constant at days 3 and 10, then was reduced to the same levels at days 21 and 35 (Figure 4G). No treatment effects were observed (*p* = 0.92) for the mRNA expression of BSEP (Figure 4H).

### 3.2. Cholic and Deoxycholic Acids

#### 3.2.1. Plasma Cholic Acid

**Figure 5:**
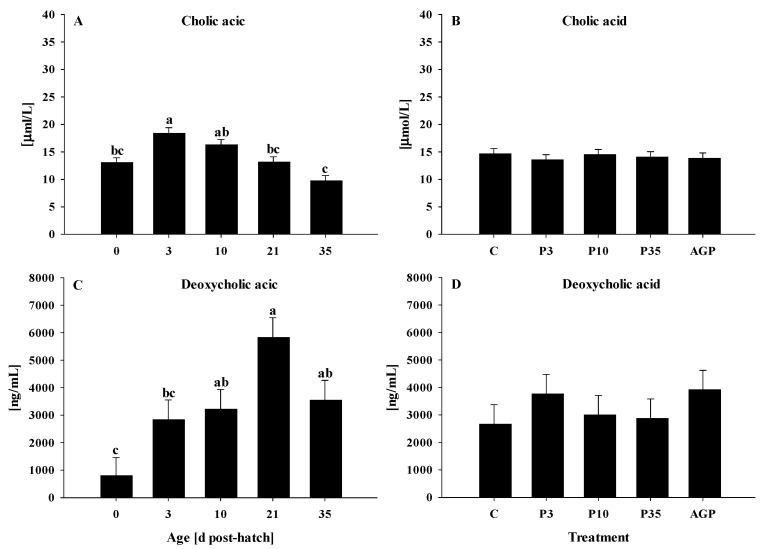
Age and treatment effects on plasma cholic acid and ileal deoxycholic acid. Age effects on (**A**) plasma cholic acid and (**C**) ileal deoxycholic acid. Treatment effects on (**B**) plasma cholic acid and (**D**) ileal deoxycholic acid. Each value represents mean ± SE. Treatments are C, normal water (without supplements) from day 0 to 35 post-hatch; P3, FM in water for first 3 days post-hatch followed by normal water until day 35; P10, FM in water for first 10 days post-hatch followed by normal water until day 35; P35, FM in water from hatch to day 35; and AGP, AGP in water from hatch to day 35. **^a,b,c^** Letters indicate significance (*p* < 0.05) between days post-hatch.

Plasma cholic acid was increased (*p* < 0.0001) from day 0 to 3 and gradually returned to day 0 level on day 35, with days 10 and 21 being intermediate (Figure 5A). No treatment differences were observed (*p* = 0.61) for the plasma cholic acid (Figure 5B).

#### 3.2.2. Ileal Deoxycholic Acid

Ileal deoxycholic acid was increased (*p* < 0.0001) from day 0 to 10 and remained elevated at days 10, 21, and 35 (Figure 5C). No treatment differences were observed (*p* = 0.92) for deoxycholic acid (Figure 5D).

## 4. Discussion

Probiotics are promoted and used as alternatives to AGPs; however, how and whether they affect the bile acid metabolism is not well understood. One of the mechanisms by which AGPs improve growth performance is the reduction of bile salt hydrolase-producing bacteria in the intestine [9,13,23]. It is well established that most lactic acid-producing bacteria possess bile salt hydrolase enzymes and can deconjugate bile salts, and this may impair lipid digestion and absorption [24]. The deconjugation of bile salts increases their excretion and subsequent hepatic bile acid neosynthesis to offset what has been lost. Previous studies showed that lactic acid-producing bacteria increased bile acid neosynthesis in chickens [25], rodent [26,27], and swine [28] models. Contrary to these reports, the supplementation of FM, made of lactic acid-producing bacteria *Lactobacillus salivarius* and *Pediococcus parvulus*, did not affect the bile acid excretion and enterohepatic circulation in the current study.

Bile acid is synthesized in the liver through the classic pathway in which CYP7A1 is the rate-limiting enzyme [8]. In the current study, the CYP7A1 mRNA level was not affected by FM, but this result is not supported by those previously reported in swine [28] and rodents [26]. The differences are likely due to species differences and probiotic types used. The current study used FM, while previous studies used *L. delbrueckii* [28] and VSL#3, a proprietary probiotic made of lactic acid-producing bacteria [26]. The lack of differences in the current study is likely because the FXR-FGF19 axis was not disturbed by the probiotic treatment. In fact, bile salts upregulate the FXR, which stimulates the release of FGF19 by enterocytes into the portal vein. In the liver, FGF19 binds to FGFR4-βklotho complex and represses CYP7A1, thereby inhibiting bile acid neosynthesis [7,29]. However, in the current study, no differences were observed in FXR, FGFR4, and βklotho. Although no differences were observed for the rest of the genes mentioned above, FGF19 was upregulated in AGP compared to P35. The probiotic was expected to deconjugate bile salt and increase bile acid neosynthesis; however, the differences observed in FGF19 expression did not affect the expression of CYP7A1, and this may explain why the FXR-FGF19 axis was not disturbed by the probiotic treatment. Although it is currently unclear why no CYP7A1 differences were observed, the data suggest that FM may not increase bile salts deconjugation and may not subsequently negatively impact dietary fat digestion and absorption in chickens. However, further studies are needed to verify this hypothesis and clarify the effects of FM on bile acid synthesis.

It is well established that about 95% of the bile acid is reabsorbed in the intestine, and the rest is excreted due to bacterial bile salt hydrolase activities as previously mentioned [8]. As a result, the liver synthesizes bile acid from cholesterol to offset the portion lost and maintain the bile acid pool [7]. In the current study, no ileal deoxycholic acid and plasma cholic acid differences were observed between FM, AGP, and C treatment groups. The reason for the lack of differences is unclear; however, it is possible that the bacteria species used in this study did not have enough bile salt hydrolase enzyme activity. FM is made of *L. salivarius* and *P. parvulus*. Although *L. salivarius* has been shown to have bile salt hydrolase activity [9,30], information on *P. parvulus* is lacking. Therefore, further research is needed to ascertain the bile salt hydrolase enzyme activity of *P. parvulus* in chickens. Nevertheless, the results of the current study disagree with previous reports on *L. salivarius* in chickens [9]. The discrepancy between the two studies could be due to the dosage and the form of probiotics. In the current study, FM, which is a combination of two bacteria, was supplemented at about 10^6^ cfu/mL, while the previous study [9] used a single strain probiotic of *L. Salivarius* that was administered to chickens by gavage at a higher dose of 10^9^ cfm/mL. Because no difference in ileal deoxycholic acid and plasma bile acid were observed in the current study, the data suggest that FM supplementation has no detrimental effects on the enterohepatic circulation.

The reabsorption and trafficking of bile salts through the enterohepatic circulation require their transport in the ileum and the liver [31]. In the ileum, ASBT is responsible for the active absorption of bile salts; I-BABP transports bile salts through the cytoplasm of enterocytes to the basolateral membrane where Ostα and Ostβ release them into the portal vein [7,32]. The sodium-dependent taurocholate protein is needed for the uptake of bile salts into the liver, while BSEP exports bile salts into the bile [7,32]. In the current study, FM supplementation did not affect any of the bile acid transport genes suggesting that the probiotic bacteria contained in the mixture did not affect the amount of bile salts reabsorbed, and this could partially explain the lack of differences observed in bile acid neosynthesis since none of the treatments increased the bile acid excretion. Limited data exist on the effects of probiotics supplementation on bile acid metabolism in chickens. Contrary to the results of the current study, in a study in which *L. delbrueckii* was fed to swine, gene expression of ileal ASBT, and I-BABP were downregulated suggesting that bile acid excretion was increased [28]. The discrepancy between the previous study and the current one may be explained by the species and probiotic types. The lack of differences in the current study suggests that FM did not deconjugate bile salts as expected. However, further study should be conducted to determine the bile salt hydrolase capacity of FM containing *L. salivarius* and *P. parvulus*.

Although no probiotic supplementation differences were observed for most of the genes, all the genes related to the bile acid metabolism were affected by age in the current study. As previously mentioned, bile salts upregulate the ileal FXR, which stimulates the release of FGF19 by enterocytes into the portal vein. In the liver, FGF19 binds to FGFR4-βklotho complex and represses CYP7A1, thereby inhibiting bile acid neosynthesis [7,32]. In the current study, although ileal FXR was upregulated at hatch and slightly downregulated during the rest of the study, FGF19 was downregulated by day 3 and was progressively upregulated until day 35 post-hatch. As a result, CYP7A1 and CYP8B1, enzymes involved in bile acid syntheses, followed the opposite pattern, and were downregulated by day 35 suggesting that the bile acid neosynthesis was upregulated at hatch and day 3 post-hatch but was reduced by day 35 post-hatch. The results are consistent with the previous report that bile acid synthesis is age dependent [33,34]. These data are not surprising because chicks are switching from lipid and cholesterol contained in the egg yolk to exogenous feed and bile acid is needed for the digestion and absorption of dietary fat and fat-soluble nutrients.

Bile acid neosynthesis offsets the loss of bile acid and ensures the maintenance of the bile acid pool through the enterohepatic circulation, which relies on different membrane-bound and cytoplasmic transporters [31]. In the current study, ASBT was downregulated at hatch but increased on the rest of the days post-hatch. This result agrees with previous research in which ASBT was downregulated at hatch but increased by day 7 post-hatch in chickens [35]. Additionally, other transporter genes I-BABP, OSTα, OSTβ, and BSEP were upregulated at hatch but decreased on day 3, suggesting that the reabsorption and trafficking of bile acid are age dependent. The upregulation of transporter genes after hatch may be an adaptation to increase bile acid reabsorption for maintaining the bile acid pool. Furthermore, plasma cholic acid and ileal deoxycholic acid were increased from hatch to days 3 and 10, respectively, confirming that the bile acid reabsorption and excretion are affected by age, as previously reported [33,34].

## 5. Conclusions

We hypothesized that FM, made of *L. salivarius* and *P. parvulus*, would increase the deconjugation and excretion of bile acid resulting in increased bile acid neosynthesis. Contrary to the hypothesis, FM did not affect bile acid neosynthesis and enterohepatic circulation. The data of the study suggest that FM did not have a negative effect as expected on the bile acid pool and may not negatively impair fat digestion and absorption due to its bacterial composition. Further study should be conducted to confirm these results and investigate the effects of FM on bile acid metabolism, fat digestion, and intestinal microbiota in chickens. In addition, the data confirm that the bile acid neosynthesis is age dependent, and the use of exogenous bile salts at different stages of poultry production should be explored to improve fat digestion.

## Figures and Tables

**Table 1:** Primers for the analysis of mRNA levels using quantitative real-time PCR.

Gene	GenBank Accession No.	Forward Primer (5’→3’)	Reverse Primer (5’→3’)	Amplicon Size (bp)
ASBT	NM_001319027.1	AAGGCTCGTGGGTTATCA	ACGACATCTGCTCCAAGA	119
BSEP	XM_025152623.1	GTTCCCACTCATTCATCCTC	TCCTCTCCCTCAGTTCATAC	97
CYP7A1	AY700578.1	TCCTCAACTGCTGCATTTTGA	GCTATTCCTGCCCCAAATGG	156
CYP8B1	NM_001005571.1	CAGGAGAGGAGAAGCAACCA	TGTTCCCTGTCCCTTGGTAC	120
FGF19	NM_204674.2	CCGCCAGCAATTCTTCTA	GCAGCGTTTGAGTCACTA	86
FGFR4	XM_015293863.2	TCATCATCGTGGTGCTGT	GTCGGATGAGTGGGAATTTG	99
FXR	AF492497.1	GAAAGGACCACACAGCAT	CTCCGTGCCAAGTTTCTA	97
I-BABP	NM_001277701.1	GTGGGATGTTTGAGTCAGTG	TCTGCTGTTCCTCTGTGA	120
SHP	AY700583.1	AGCATGCTCGAGAAGATCCT	GCTCAAATCCAGGCTCCAGA	129
OSTα	NM_001277697.1	GAAACCAAGGCAGTCAGT	ATCATCTGCCAGCTCCAT	88
OSTβ	XM_025153901.1	GAGGAGAAAGCAGCACAA	CCAGCACAAGGACATCAT	94
βKlotho	XM_003641245.5	GGCCTCTCACACTCTTCACT	CTCATACTGGCTCCCGTTCT	138
GAPDH	NM_204305	AGCCATTCCTCCACCTTTGAT	AGTCCACAACACGGTTGCTGTAT	112
β-actin	X0082	TTCTTTTGGCGCTTGACTCA	GCGTTCGCTCCAACATGTT	88
β2-m	Z48921	TGGAGCACGAGACCCTGAAG	TTTGCCGTCATACCCAGAAGT	161

Abbreviations: ASBT, apical sodium-dependent bile acid transporter; BSEP, bile salt exporter pump; CYP7A1, cholesterol 7 alpha-hydroxylase; CYP8B1, sterol 12 alpha-hydroxylase; FGF19, fibroblast growth factor 19; FGFR4, fibroblast growth factor receptor 4; FXR, farnesoid X receptor; I-BABP, ileal bile acid binding protein; SHP, small heterodimer partner; OSTα, organic solute transporter alpha; OSTβ, organic solute transporter beta; βklotho, beta klotho; GAPDH, glyceraldehyde-3-phosphate dehydrogenase; β-actin, beta-actin; and β2-m, beta2-microglobulin.

## Data Availability

The data that support the findings of this study are available from the corresponding author, K.R.K., upon reasonable request.
